# Exposure to Volatile Organic Compounds in Paint Production Plants: Levels and Potential Human Health Risks

**DOI:** 10.3390/toxics11020111

**Published:** 2023-01-24

**Authors:** Safiye Ghobakhloo, Amir Hossein Khoshakhlagh, Simone Morais, Ashraf Mazaheri Tehrani

**Affiliations:** 1 Department of Environmental Health Engineering, School of Health, Kashan University of Medical Sciences, Kashan 8715988141, Iran; 2 Department of Occupational Health Engineering, School of Health, Kashan University of Medical Sciences, Kashan 8715988141, Iran; 3 REQUIMTE-LAQV, Instituto Superior de Engenharia do Porto, Instituto Politécnico do Porto, Rua Dr. António Bernardino de Almeida, 431, 4249-015 Porto, Portugal

**Keywords:** paint production plant, volatile organic compounds (VOCs), inhalation exposure, cancer risk, non-cancer risk

## Abstract

A wide range of volatile organic solvents, including aliphatic and aromatic hydrocarbons, alcohols, and ketones, are used in the production of paints, and they comprise more than 30% of the ingredients of paints. The present study was designed to evaluate the occupational exposure to 15 volatile organic compounds (VOCs, including benzene, toluene, ethylbenzene, xylene, styrene, n-hexane, n-heptane, n-nonane, trichloroethylene, tetrachloroethylene, n-butyl acetate, n-octane, n-decane, dichlorofluoromethane, and acetone) in Iranian paint production factories and subsequently, the associated health risks. The samples were collected from the respiratory zone of workers using the NIOSH 1501 method, and their qualitative and quantitative characterization was performed using gas chromatography-mass spectrometry and gas chromatography-flame ionization detector, respectively. The individual concentrations of VOCs ranged from 23.76 ± 0.57 µg m^−3^ (acetone) to 92489.91 ± 0.65 µg m^−3^ (m,p-xylene). The predominant compounds were m,p-xylene (up to 92489.91 ± 0.65 µg m^−3^), ethylbenzene (up to 91188.95 ± 0.34 µg m^−3^), and toluene (up to 46088.84 ± 0.14 µg m^−3^). The non-cancer risks of benzene, n-nonane, trichloroethylene, tetrachloroethylene, xylene, and ethylbenzene surpassed the reference value in most of the sectors. In addition, total lifetime risks of cancer were in the range of 1.8 × 10^−5^–3.85 × 10^−3^, suggesting that there was a risk of carcinogenesis in all studied sections, mainly due to ethylbenzene and benzene. Considering their high exposure concentrations and their associated non-carcinogenic and carcinogenic risks, biological monitoring of workers and the use of technical and modern engineering control measures are recommended.

## 1. Introduction

Volatile organic compounds (VOCs) are a wide variety of chemical substances that are derived from natural processes and human activities [1]. In the occupational context, these compounds are widely used in industrial processes, such as rubber manufacturing, plastic manufacturing, paint production, and automobile manufacturing [2,3,4]. Benzene, toluene, ethylbenzene, and xylene, which are among the most common VOCs, are known to pose risks to human health [5,6]. In addition to hydrocarbons, halocarbons and oxygenated hydrocarbons, such as styrene (vinyl benzene), are also classified as harmful compounds to human health. Styrene is an economically industrial chemical that is utilized in the synthesis and manufacturing of polystyrene and hundreds of different copolymers, as well as in many other industrial resins. Short-term exposure to high concentrations of VOCs may irritate the eyes, nose, throat, and lungs, as well as damage the liver, kidneys, and central nervous system. Additionally, long-term exposure to low concentrations of pollutants can lead to asthma, reduced lung function, cardiovascular disease, and cancer [7]. The International Agency for Research on Cancer (IARC) and the United States Environmental Protection Agency (USEPA) have classified benzene as a known human carcinogen (Group A), ethylbenzene and styrene as possibly carcinogenic to humans (Group 2B), and tetrachloroethylene and trichloroethylene as probable carcinogens to humans (Group 2A) [8]. Many studies have shown that inhalation is the main route of exposure to VOCs [9,10], and that significant risks for workers of different industries (gas station workers, tire-manufacturing factories, and dyeing industrial complex, among others) may exist [6,11,12,13]. Considering the potential toxic effects of VOCs on people’s health in work environments, monitoring these compounds and assessing their health risks is the first way to adopt control measures for occupational exposure and regulatory purposes at the national and international level [14]. There are different methods to determine exposure to chemicals in work environments, and the direct measurement of pollutant concentration in a person’s respiratory area is considered the most reliable method. By combining data related to exposure and the dose-response of the chemicals, risks from exposure to chemicals can be calculated [15]. Hu et al. found that the lifetime cancer risks of benzene, tetrachloromethane, trichloromethane, and trichloroethylene in different functional zones (traffic, industrial, development, resident, and ground zone) of a typical developing city in China were above the acceptable risk level (1.0 × 10^−6^) set by USEPA [16]. Shuai et al. reported that the prevalence of respiratory, allergic, and cardiovascular diseases near the dyeing industrial complex in South Korea was significantly higher than in the control area [12]. The results of Tunsaringkarn et al. showed that occupational exposure to BTEX increased the risk of cancer in gas station workers [6]. Hosseini et al. reported unacceptable occupational cancer risks due to benzene exposure in two tire-manufacturing factories [11]. Other similar studies indicated significant risks of VOC exposure in different occupational and non-occupational environments [17,18,19,20]. Because organic solvents are still one of the main constituents of paints, workers from the paint and painting industries are regularly and occupationally exposed to them [21]. Golbabai et al. showed that the carcinogenic risks for benzene and ethylbenzene and the non-cancer risks for benzene and xylene in the paint section of an automotive industry were higher than the recommended level [13]. On the other hand, the market has been moving towards industrial paint applications in industries such as construction, automotive, general, coils, wood, aerospace, fences, and packaging coatings, which leads to the growth of demand [22]. Considering that the workforce is considered the capital of every society, providing, maintaining, and improving their health is one of the most important goals of every society [23]. Thus, in this study, and due to the limited information on the subject, the USEPA model [24,25] was used to assess the health risks of VOC exposure (to 15 compounds, including benzene, toluene, ethylbenzene, m/p- xylene, styrene, n-hexane, n-heptane, n-nonane, trichloroethylene, tetrachloroethylene, n-butyl acetate, n-octane, n-decane, dichlorofluoromethane, and acetone) in paint factories of Iran during 2022.

## 2. Materials and Methods

### 2.1. Site Description

This cross-sectional study was conducted on workers (all male) from two paint plants in the Semnan province of Iran in 2022. The characterized production processes were those conventionally used in Iran and took place on two closed floors of the plant. The workers of the production line were classified according to job operations. These people worked in units called raw materials, mixing and dispersion, and filling lines. Workers in the raw materials line began the process by emptying the paint materials into tanks connected to buckets in the mixing and dispersing department by pipes. In the second part, colored liquors were mixed with porcelain clays. The paint then went to the filling line and was emptied into cans for sealing and shipping. The manufacturing process was maintained, and air from the respiratory zone of the workers was collected for 8 h during the working shift. A temperature of 80 °C produced significant paint fumes in the work area. The paint production unit of the studied industry had 8 sections, including 3 different paint production salons [Plastic Color production (PC), Cathodic Electrodeposition production (CED), and Original Equipment Manufacturer Color production (OEM)], 2 paint warehouses (dispatch and topcoat), a washing salon (washing PC salon), and 2 paint laboratories (lab OEM and PC lab).

### 2.2. Sampling Method

The NIOSH-1501 method was used to assess the occupational exposure. None of the workers used personal protective equipment (PPE), including facemasks and protective clothing. VOC samples were collected in each factory section using tubes containing solid adsorbents of activated carbon (SKC Inc., Pittsburgh, PA, USA) and an individual sampling pump, calibrated at a flow rate of 200 mL min^−1^ (SKC Inc., Pittsburgh, PA, USA). Sampler specifications included a glass tube with a length of 7 cm, an inner diameter of 6 mm OD, and an outer diameter of 4 mm ID and flamed sealed ends with plastic caps containing two sections of 20/40 mesh-activated (600 °C) coconut shell charcoal (front = 100 mg, back = 50 mg) separated by a 2 mm urethane foam plug. A total of 75 individual samples of air were collected during sampling, and the environmental factors, such as temperature, humidity, air pressure, airflow speed, and the condition of the existing ventilation system on the concentration of pollutants in the workplace air were recorded.

### 2.3. Sample Preparation and Analysis

After collection, the samples were transported to the laboratory. Both the front and back sections of the activated carbon tube were transferred to different 2 mL vials. Samples were extracted, with 1 mL carbon disulfide (99.5%) (Merck Inc., Darmstadt, Germany) as eluent under ultrasonic waves for at least 30 min to complete extraction. Qualitative information about the predominant VOCs was obtained by gas chromatography-mass spectrometry (6890N/5973; Agilent, Palo Alto, Santa Clara, CA, USA). Analysis was performed using gas chromatography (GC 7890 Agilent, Santa Clara, CA, USA) equipped with a flame ionization detector (FID) using a capillary column (length = 30 m, internal diameter = 0.25 mm). Helium gas was used as a carrier gas, with a flow rate of 2 mL min^−1^. The injection volume was 1 μL, and a split ratio of 5/1 was applied. The initial temperature of the column was 50 °C, which increased to 100 °C after 5 min. The injector was set at a temperature of 250 °C. Standard solutions of benzene, ethylbenzene, xylene, toluene, styrene, n-hexane, n-heptane, n-nonane, trichloroethylene, tetrachloroethylene, n-butyl acetate, n-octane, n-decane, dichlorofluoromethane, and acetone (Merck Inc., Darmstadt, Germany) were used to obtain the calibration curves.

### 2.4. Quality Control (QC)/Quality Assurance (QA)

In this study, the concentration of BTEX compounds was read according to the ISO/IEC 17025 standard method using the carbon disulfide extraction method and a gas chromatograph (GC) coupled with an FID in the laboratory. In this method, the detection limits for VOCs were in the range of 0.04 and 30 µg m^−3^ (for a sample preconcentration of 1 m^3^) [26]. Additionally, control samples and duplicate samples (obtained from all study sites) were used. The relative deviation of all VOCs in duplicate samples was less than 11%. Five blank samples were taken to check the presence of any possible contamination during the sampling, transportation, and storage of air samples. In this study, the total concentration of VOCs in each blank sample was found to be <0.5 ppbv. Spiked samples were used to assess the recovery rate and accuracy. Accuracy and precision were determined by analyzing 15 replicates of QC samples on three different days. The results showed that the analyte recovery percentage was >95% for most compounds.

### 2.5. Health Risk Assessment

The cancer risk assessment for benzene, ethylbenzene, styrene, trichloroethylene, and tetrachloroethylene and the non-carcinogenic health risk assessment for all VOCs were performed using the EPA method [24,25]. After determining the concentration of pollutants, the adjusted air exposure concentration (EC, mg m^−3^) was calculated in order to represent the duration of exposure through Equation (1), based on USEPA recommendations [25].

EC (mg m^−3^) = (C × ET × EF × ED/AT) (1)

where C (mg m^−3^) is the concentration of the considered compound in the collected personal air sample; ET (h day^−1^) is the exposure time per day; EF (days year^−1^) is the exposure frequency per year; ED (years) is the exposure duration; and AT (hours) is the average lifetime (Table 1).

The hazard quotient (HQ) index was calculated to estimate the potential risk posed by the non-carcinogenic effects of the chemical compounds (Equation (2)). The total hazard quotient (THQ) is the sum of the individual HQs.

Hazard Quotient (HQ) = EC (mg m^−3^)/RFC (mg m^−3^) (2)

where RFC is the reference concentration for inhalation exposure (Table 2).

The chronic daily intake (CDI) was calculated by:

CDI (mg kg^−1^ day^−1^) = (C × IR × EF × ED/LT × BW) (3)

where BW is the body weight (kg), IR is the inhalation rate (m^3^ day^−1^), and LT is the lifetime (day) (Table 1).

If the lifetime risk of cancer (LTCR; Equation (4)) was less than or equal to one in a million (1 × 10^−6^), it had no significant effects on human health, so cancer risk was negligible. A LTCR more than 1 × 10^−4^ was established as “definite risk,” between 1 × 10^−4^ and 1 × 10^−6^ as “probable risk,” between 1 × 10^−5^ and 1 × 10^−6^ as “possible risk,” and less than 1 × 10^−6^ as “negligible risk” for human health [27]. The cancer slop factor (CSF) for benzene, ethylbenzene, styrene, trichloroethylene, and tetrachloroethylene are shown in Table 2 [10].

LTCR = CDI (mg kg^−1^ day^−1^) × CSF [(mg kg^−1^ day^−1^)]^−1^ (4)

**Table 1:** Information for risk assessment.

Parameter	Values	Data Collection
Exposure time to VOCs (hours/days)—ET	8	Questionnaire
Exposure frequency (day/year)—EF	300	Questionnaire
Exposure duration(years)—ED	30	USEPA, 2002 [25]
Lifetime (day)—LT	25,600	USEPA, 2011 [28]
Inhalation rate (m^3^ day^−1^)—IR	16	USEPA, 2011 [25]
Body weight (kg)—BW	72 ± 9.42	Questionnaire
Average lifetime (hours)—AT	33,650	USEPA, 2011 [28]

**Table 2:** Inhalation dose reference exposure (RFC) and cancer inhalation unit risk for the characterized VOCs.

Agent	RFC (mg m^−3^)	Cancer Slop Factor (mg kg^−1^ day^−1^)	USEPA/IARC Class	Reference
Benzene	0.03	0.029	A	IRIS ^a^
Toluene	5	…		IRIS
Ethylbenzene	1	0.0087	2B	IRIS
m,p-Xylene	0.1	…		IRIS
Styrene	1	5.7 × 10^−4^	2B	CEP ^b^
n-Hexane	0.7	…		IRIS
n-Heptane	0.4	…		IRIS
n-Nonane	0.02	…		IRIS
Trichloroethylene	0.002	1.1 × 10^−2^	2A	IRIS
Tetrachloroethylene	0.04	2.07 × 10^−2^	2A	IRIS
n-Butyl acetate	1.429	…		WHO ^c^
n-Octane	1.111	…		MHLW ^d^
n-Decane	0.836	…		Sagunski and Mangelsdorf [29]
Dichlorofluoromethane	0.330	…		IRIS
Acetone	56	…		OECD ^e^

^a^ IRIS: Integrated Risk Information System from USEPA. ^b^ CEP: Cumulative Exposure Project from USEPA. ^c^ WHO: World Health Organization. ^d^ MHLW: Ministry of Health, Labor, and Welfare. ^e^ OECD: Organization for Economic Co-operation and Development.

## 3. Statistical Analysis

The analysis results of VOCs were expressed as mean ± standard deviation using SPSS 22 (Chicago, IL, USA). One-way analysis of variance (ANOVA) was used to determine the difference between the average exposure to VOCs in different units. The relationship between the data was checked at a significance level of 0.05.

## 4. Result and Discussion

### 4.1. Levels of the VOCs in the Personal Air in the Paint Factories

**Figure 1:**
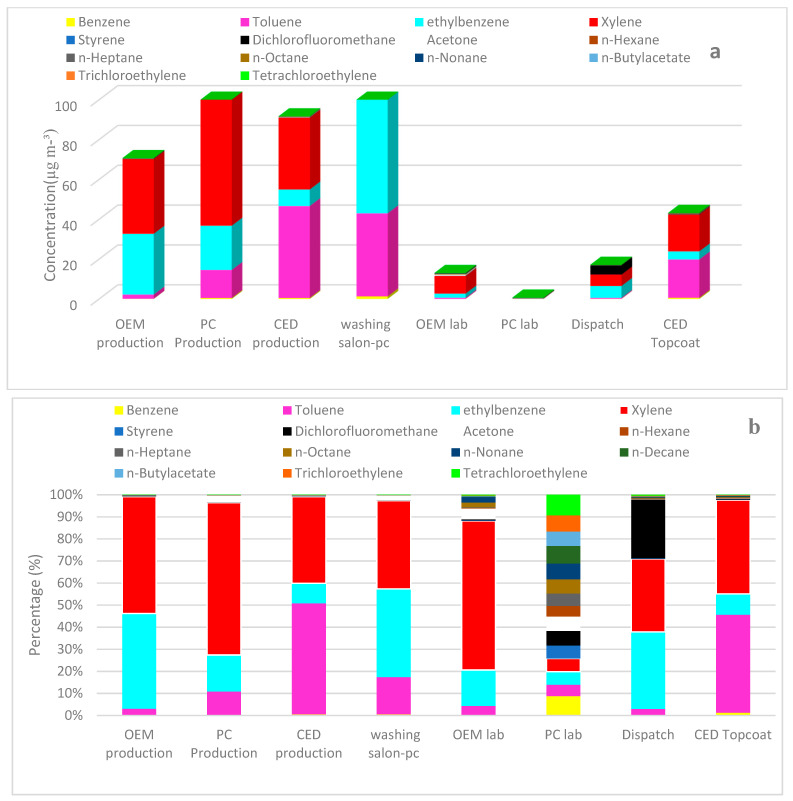
Comparison of (**a**) concentrations and (**b**) percentages of the characterized VOCs in different sectors of the paint factories.

Based on the results of the qualitative analysis of the gas chromatography-mass spectrometric detection, 15 compounds were identified and quantified by GC-FID (Table 3). The concentrations of VOCs ranged from 23.76 ± 0.57 (dispatch) to 92489.91 ± 0.65 µg m^−3^ (production). The analysis of VOCs showed that the decreasing order of the total concentrations of VOCs detected was the washing salon-pc ≫ PC production > CED production, the three of them being identified as the most polluted areas. The most abundant compounds, in order, were xylene (5.95% to 69.03%) > toluene (2.98% to 50.26%) > ethylbenzene (5.94% to 43.14%) (Figure 1). The maximum values detected for xylene (92,489.91 ± 0.65 µg m^−3^ and 81,200.06 ± 0.45µg m^−3^ in the PC production and washing salons, respectively) and ethylbenzene (91,188.95 µg m^−3^, 21.07 ppm in the paint-washing PC salon) exceeded the occupational exposure limit of 20 ppm provided by the Environmental and Occupational Health Center of Iran (EOHCI). The results obtained in this study are in line with those reported for other countries. Mo et al. [10] conducted a study to assess the human health risk of VOCs in the paint and coatings industry in the Yangtze River Delta, China. They found that toluene, m/p-xylene, and ethylbenzene were the prevalent compounds in the container coating sector (22.01%, 23.11%, and 17.73%, respectively), ship coating sector (28.73%, 22.76%, and 25.78%, respectively), and furniture coating sector (13.40%, 27.5%, and 27.16%, respectively) [10]. Omidi et al. reported that benzene concentrations in the energy, biochemical, and benzol refining sectors from Iran were higher than the set national occupational exposure limit, opposite to the levels of toluene, ethylbenzene, and xylene in other studied sectors (muffle furnace, battery, and material recycling) [30]. Additionally, Dehghani et al. reported benzene concentrations up to 3.035 mg m^−3^ (equivalent to 0.95 ppm) in the paint cabin section, which surpassed the occupational exposure limit (0.5 ppm) provided by the Environment and Labor Health Center of the Ministry of Health [31].

### 4.2. Health Risk Assessment

**Figure 2:**
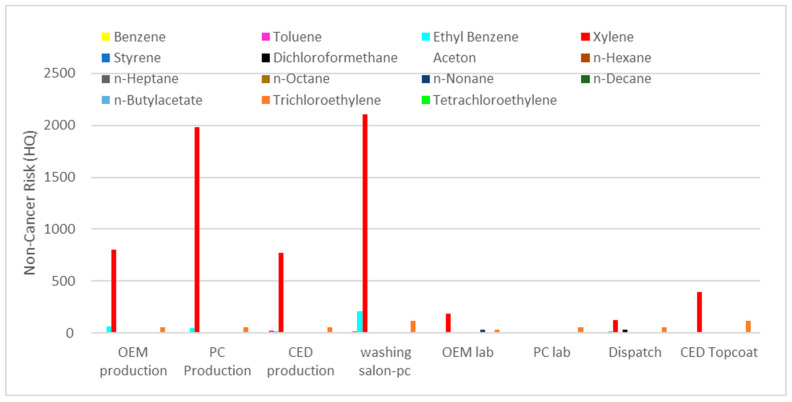
Comparison of non-cancer risk in different sectors of the factories.

The data of EC, HQ, and CDI of the characterized VOCs in different parts of the factory are displayed in Table 4 and Figure 2 [32].

HQ ≤ 1 indicates that adverse health effects are unlikely to occur, whereas HQ > 1 means that there may be risks to sensitive individuals as a result of exposure. Sectors with relatively high non-cancer risk values and their exposed workers were identified. The non-cancer risk values of benzene, n-nonane, trichloroethylene, and tetrachloroethylene in all parts of the factory exceeded the safe level of one. Additionally, the non-cancer risk values of xylene, ethylbenzene, and toluene surpassed the reference value in most of the sectors, with the PC lab being the common safer site (HQ < 1). The non-cancer risks were higher in washing salon-PC, followed by production salon-PC, OEM salon, CED production, CED topcoat, OEM lab, dispatch, and PC lab. On the contrary, all the other compounds, i.e., styrene, dichlorofluoromethane, acetone, n-Hexane, n-heptane, n-octane, n-decane, and n-butylacetate within several sectors exhibited acceptable non-carcinogenic risks (HQ < 1). However, exposure to multiple hazardous pollutants may promote combined and/or synergistic effects. Possible associations were suggested between exposure to chlorinated solvents (such as tetrachloroethane, trichloroethylene, and tetrachloroethylene), benzene, lead, and asbestos and the risk of breast cancer in women (exposed workers) [33].

Table 5 presents the total and individual carcinogenic risks of the VOCs in the selected paint factories. USEPA considers the acceptable risk level to be in the range of 1 × 10^−6^ to 1 × 10^−4^. For carcinogens, USEPA considers excess cancer risks that are below 1 chance in 1,000,000 (1 × 10^−6^) to be so small as to be negligible. However, for a residual cancer risk of less than 10^−4^, it is recommended to ensure that there is no cumulative cancer risk of potentially carcinogenic compounds. According to the results of Table 5, total LTCR values were in the range of 1.8 × 10^−5^–3.85 × 10^−3^, suggesting that there was a risk of carcinogenesis in all studied sections. The cancer risk of ethylbenzene was higher than 1 × 10^−4^ in all sectors of the factories except in PC lab, while the cancer risks of tetrachloroethane and trichloroethylene were lower than 1 × 10^−5^ in all sectors of the factories. The cancer risk of styrene was higher than 1 × 10^−6^ only in the CED topcoat sector. The exposure to benzene presented cancer risks in the range of 1.99 × 10^−5^ to 7.94 × 10^−4^. Thus, ethylbenzene was the predominant contributor to the determined increased risk of cancer. The washing salon-PC, CED production, dispatch, OEM production salon, PC production salon, CED topcoat, and OEM lab were the most polluted environments, with the highest risk of cancer being for ethylbenzene (5.28 × 10^−2^, 4.47 × 10^−3^, 3.21 × 10^3^, 1.64 × 10^−3^, 1.19 × 10^−3^, 2.19 × 10^−4^, and 1.14 × 10^−4^, respectively). This means that workers in these sectors may suffer from a cancer risk 45–530 times higher than 1 additional case per 10,000 employees exposed (1 × 10^−4^), i.e., the upper limit of acceptable cancer risk (1 × 10^−4^) established by USEPA recommendations. These findings emphasize the role of ethylbenzene compounds in the occupational exposure in the paint industry in Iran. One of the reasons for the high level of ethylbenzene in this section is the presence of ethylbenzene impurity in the solvents and the excessive use of thinner in cleaning surfaces, despite the elimination/reduction in many raw materials. These data are consistent with the results reported by Golbabaei et al. [13]. It was also found that ethylbenzene in spray paints (9.71 × 10^−4^), wooden furniture manufacturing (1.75 × 10^−5^), municipal solid waste (1.71 × 10^−6^), electronic waste dismantling processes (6.2 × 10^−3^), the rubber footwear industry (>1 × 10^−4^), and the oil refinery (6.09 × 10^−3^) originates high cancer risks [10]. In addition, considering the other determined VOCs, the only exceedance was detected for the LTCR of benzene in washing salon-PC (1.64 × 10^−4^), which was in agreement with the reported information from Zhang et al. and Chen et al., who found average LTCR values of 3.4 × 10^−4^ and 4.1 × 10^−5^, respectively, in the ambient air of Beijing, China and the petrochemical industrial complexes [34,35]. Benzene contributes significantly to the risk of cancer in petrochemical industries, rubber shoes, asphalt paving, and coking wastewater treatment industries [36,37], supporting its selection in this study. Exposure to benzene may cause a potential risk of adverse health effects during a thirty-year exposure period. Lan et al. [38] conducted a study to assess the risk of benzene in three clothes-manufacturing factories in the same region near Tianjin. Despite benzene levels being lower than the permissible limits, the relative risk of leukemia for employees was reported to be 1.1 times higher than in the non-exposed group.

During working hours, workers are exposed to various hazards, including contact with chemicals, biological and physical factors, and unfavorable ergonomic conditions, which are responsible for a variety of health outcomes [39]. Firoozeh et al. [40] found that chronic occupational exposure to excess amounts of mixed organic solvents can cause decreased motivation and mental fatigue in exposed individuals. The results of a study at a petrochemical plant in China showed that xylene, benzene, and toluene are potentially involved in causing lung dysfunction. Physiologically based pharmacokinetic results showed that the metabolism of ethylbenzene was strongly reduced by simultaneous exposure to high concentrations of xylene, leading to non-linear behavior [41]. Additionally, in recent years, various studies have been conducted to assess the health risk of exposure to organic solvents in paint factories. A cross-sectional study involving 97 workers from a paint plant in Mexico showed a significant association between macrocytosis and exposure to high doses of BTX mixtures (OR: 3.6, 95% CI: 1.08 to 13.9, *p* = 0.02) [42]. Hassan et al. found that neuropsychological symptoms were 63.04% in paint manufacturing workers, while it was only 2.1% in the control group. Additionally, the risk of neurological symptoms was higher in the production group than in the packaging group (OR = 13.94) [43]. Ikegwuonu et al. [44] showed that the serum levels of aspartate transaminase, alkaline phosphatase, sodium, and chloride in workers working in paint plants were significantly higher than in workers working in non-paint factories. Exposure to VOCs and heavy metals in the paint plant makes workers prone to liver and kidney disorders [45].

## 5. Conclusions and Implications

This study collected VOC samples in the respiratory zone of workers in paint factories under normal occupational conditions. Xylene, toluene, and ethylbenzene were the most abundant compounds in the production processes, which was generally consistent with previous, related studies. A total of 15 VOCs were selected to evaluate their non-carcinogenic and carcinogenic risks to workers from different sectors in paint factories. The highest concentration of total VOCs was observed in the washing salon-PC sector. Non-carcinogenic risks promoted by exposure to benzene, n-nonane, trichloroethylene, tetrachloroethylene, xylene, and ethylbenzene were found almost in all of the sectors of the factories. For carcinogens, the LTCR values significantly exceeded the value of the negligible risks, which was 1.0 × 10^−6^. Ethylbenzene and benzene were the most critical pollutants that contributed to the high risk of cancer in these factories. Considering the high exposure concentrations and the high non-carcinogenic and carcinogenic risks of these compounds, the use of PPE, biological monitoring of workers, and the use of technical and modern engineering control measures are highly recommended. Additionally, in order to reduce VOC emissions directly at the source, paints with low VOC content or without VOCs (new environmentally friendly paints) are urgently needed.

## Figures and Tables

**Table 3:** Mean concentrations of the VOCs detected in several sections of the characterized paint factories.

^a^ VOCs	^b^ TLV-TWA (ppm)	Mean ± SD (µg m^−3^) (%)							
		^c^ OEM Production	^d^ Production	^e^ CED Production	Washing Salon-PC	^f^ OEM Lab	^g^ PC Lab	Dispatch	^h^ CED Topcoat
Benzene ^i^ (LOD = 0.5)	0.5	63.89 ± 0.27 (0.09%)	447.25 ± 0.21 (0.33%)	479.20 ± 0.23 (0.52%)	1277.87 ± 0.32 (0.51%)	31.94 ± 0.08 (0.24%)	63.89 ± 0.65 (8.75%)	63.89 ± 0.17 (0.37%)	543.09 ± 0.73 (1.25%)
Toluene (LOD = 0.7)	20	2110.36 ± 0.53 (2.98%)	14,056.53 ± 0.56 (10.49%)	46,088.84 ± 0.14 (50.26%)	37,873.49 ± 0.13 (16.87%)	527.59 ± 0.34 (4.04%)	37.68 ± 0.43 (5.16%)	452.22 ± 0.56 (2.63%)	19,219.3 ± 0.41 (44.41%)
Ethylbenzene (LOD = 0.5)	20	30,526.58 ± 0.18 (43.14%)	22,232.73 ± 0.32 (16.59%)	8337.27 ± 0.57 (9.09%)	91,188.95 ± 0.34 (39.95%)	2127.74 ± 0.45 (16.31%)	43.42 ± 0.75 (5.94%)	5992.41 ± 0.17 (34.85%)	4081.79 ± 0.37 (9.43%)
m,p-Xylene (LOD = 0.7)	20	37,473.61 ± 0.38 (52.95%)	92,489.91 ± 0.65 (69.03%)	35,997.24 ± 0.45 (39.260%)	81,200.06 ± 0.45 (39.95%)	8814.76 ± 0.76 (67.57%)	43.42 ± 0.33 (5.95%)	5688.34 ± 0.63 (33.09%)	18,411.1 ± 0.18 (42.55%)
Styrene (LOD = 0.4)	10	42.59 ± 0.42 (0.06%)	42.59 ± 0.12 (0.03%)	42.59 ± 0.24 (0.05%)	85.19 ± 0.26 (0.03%)	38.33 ± 0.18 (0.29%)	42.59 ± 0.34 (5.84%)	38.33 ± 0.69 (0.22%)	85.19 ± 0.58 (0.22%)
n-Hexane (LOD = 0.4)	50	35.24 ± 0.17 (0.05%)	35.24 ± 0.68 (0.03%)	70.49 ± 0.27 (0.08%)	105.74 ± 0.18 (0.04%)	35.24 ± 0.38 (0.27%)	35.24 ± 0.19 (4.83%)	35.24 ± 0.13 (0.21%)	105.74 ± 0.65 (0.24%)
n-Heptane (LOD = 0.06)	400	40.98 ± 0.14 (0.06%)	40.98 ± 0.23 (0.03%)	40.98 ± 0.18 (0.04%)	81.97 ± 0.61 (0.03%)	40.98 ± 0.37 (0.31%)	40.98 ± 0.23 (5.61%)	36.88 ± 0.25 (0.21%)	81.97 ± 0.23 (0.19%)
n-Nonane (LOD = 0.04)	200	52.43 ± 0.23 (0.07%)	52.43 ± 0.13 (0.04%)	52.43 ± 0.11 (0.06%)	104.86 ± 0.76 (0.04%)	314.6 ± 0.78 (2.41%)	52.43 ± 0.12 (7.18%)	41.94 ± 0.27 (0.24%)	104.86 ± 0.28 (0.24%)
Trichloroethylene (LOD = 0.6)	10	53.73 ± 0.23 (0.08%)	53.73 ± 0.16 (0.04%)	53.73 ± 0.23 (0.06%)	53.73 ± 0.43 (0.04%)	53.73 ± 0.17 (0.21%)	53.73 ± 0.52 (7.36%)	53.73 ± 0.48 (0.31%)	107.47 ± 0.56 (0.25%)
Tetrachloroethylene (LOD = 2)	25	67.82 ± 0.47 (0.10%)	67.82 ± 0.73 (0.05%)	47.47 ± 0.24 (0.05%)	67.82 ± 0.23 (0.03%)	67.828 ± 0.27 (0.36%)	67.82 ± 0.33 (9.29%)	67.828 ± 0.27 (0.39%)	33.91 ± 0.15 (0.08%)
n-Butyl acetate (LOD = 0.9)	150	47.51 ± 0.29 (0.07%)	47.51 ± 0.39 (0.04%)	47.51 ± 0.19 (0.05%)	47.51 ± 0.21 (0.02%)	28.51 ± 0.21 (0.22%)	47.51 ± 0.19 (6.51%)	28.51 ± 0.2 (0.17%)	47.51 ± 0.24 (0.11%)
n-Octane (LOD = 0.3)	300	46.72 ± 0.76 (0.07%)	46.72 ± 0.21 (0.03%)	46.72 ± 0.28 (0.05%)	93.44 ± 0.81 (0.04%)	280.32 ± 0.38 (2.15)	46.72 ± 0.52 (6.40%)	37.38 ± 0.84 (0.22%)	93.44 ± 0.19 (0.22%)
n-Decane (LOD = 0.06)	45	58.20 ± 0.65 (0.08%)	58.20 ± 0.13 (0.04%)	58.20 ± 0.72 (0.06%)	116.39 ± 0.33 (0.05%)	40.74 ± 0.73 (0.31%)	58.20 ± 0.84 (7.97%)	400.29± 0.12 (0.24%)	116.39 ± 0.14 (0.27%)
Dichlorofluoromethane (LOD = 30)	1000	97.65 ± 0.82 (0.14%)	97.65 ± 0.95 (0.07%)	97.65 ± 0.27 (0.11%)	146.48 ± 0.38 (0.06%)	48.83 ± 0.17 (0.37%)	48.83 ± 0.79 (6.69%)	4589.66 ± 0.29 (26.70%)	146.48 ± 0.48 (0.34%)
Acetone (LOD = 20)	500	47.52 ± 0.21 (0.07%)	4205.29 ± 0.64 (3.14%)	237.59 ± 0.84 (0.26%)	5702.09 ± 0.46 (2.31%)	641.48 ± 0.33 (4.92%)	47.52 ± 0.25 (6.51%)	23.76 ± 0.57 (0.14%)	95.03 ± 0.52 (0.22%)

^a^ VOCs: volatile organic compounds. ^b^ TLV-TWA (ppm): threshold limit value–Time-Weighted Average. ^c^ OEM production: original equipment manufacturer color production. ^d^ PC production: plastic color production. ^e^ CED production: cathodic electro deposition production. ^f^ OEM lab: original equipment manufacturer color laboratory. ^g^ PC lab: plastic color laboratory. ^h^ CED topcoat: cathodic electro deposition topcoat. ^i^ Limit of detection (μg m^−3^).

**Table 4:** The exposure concentrations, hazard quotients (HQ), and chronic daily intakes of VOCs through inhalation in the characterized production zones.

Pollutant	OEM Salon	Production Salon-PC	CED Production	Washing Salon-PC	OEM Lab	PC Lab	Dispatch	CED Topcoat
**Exposure concentration (mg m^−3^)**								
Benzene	0.137	0.957	1.025	2.734	0.068	0.137	0.137	1.162
Toluene	4.515	30.076	98.615	89.100	1.129	0.081	0.968	41.123
Ethylbenzene	65.317	47.571	17.839	210.910	4.553	0.093	12.822	8.734
Xylene	80.181	197.898	77.022	210.906	18.861	0.093	12.171	39.394
Styrene	0.091	0.091	0.091	0.182	0.082	0.091	0.082	0.182
Dichlorofluoromethane	0.209	0.209	0.209	0.313	0.104	0.104	9.820	0.313
Acetone	0.102	8.998	0.508	12.201	1.373	0.102	0.051	0.203
n-Hexane	0.075	0.075	0.151	0.226	0.075	0.075	0.075	0.226
n-Heptane	0.088	0.088	0.088	0.175	0.088	0.088	0.079	0.175
n-Octane	0.100	0.100	0.100	0.200	0.600	0.100	0.080	0.200
n-Nonane	0.112	0.112	0.112	0.224	0.673	0.112	0.090	0.224
n-Decane	0.125	0.125	0.125	0.249	0.087	0.125	0.087	0.249
n-Butylacetate	0.102	0.102	0.102	0.102	0.061	0.102	0.061	0.102
Trichloroethylene	0.115	0.115	0.115	0.230	0.057	0.115	0.115	0.230
Tetrachloroethylene	0.145	0.145	0.102	0.145	0.102	0.145	0.145	0.073
**Hazard quotient**								
Benzene	4.56	31.90	34.18	91.14	2.28	4.56	4.56	38.73
Toluene	0.90	6.02	19.72	17.82	0.23	0.02	0.19	8.22
Ethylbenzene	65.32	47.57	17.84	210.91	4.55	0.09	12.82	8.73
Xylene	801.81	1978.98	770.22	2109.06	188.61	0.93	121.71	393.94
Styrene	0.09	0.09	0.09	0.18	0.08	0.09	0.08	0.18
Dichlorofluoromethane	0.63	0.63	0.63	0.95	0.32	0.32	29.76	0.95
Acetone	0.00	0.16	0.01	0.22	0.02	0.00	0.00	0.00
n-Hexane	0.11	0.11	0.22	0.32	0.11	0.11	0.11	0.32
n-Heptane	0.22	0.22	0.22	0.44	0.22	0.22	0.20	0.44
n-Octane	0.12	0.12	0.12	0.24	0.72	0.12	0.10	0.24
n-Nonane	5.61	5.61	5.61	11.22	33.66	5.61	4.49	11.22
n-Decane	0.15	0.15	0.15	0.30	0.10	0.15	0.10	0.30
n-Butylacetate	0.07	0.07	0.07	0.07	0.04	0.07	0.04	0.07
Trichloroethylene	57.49	57.49	57.49	114.98	28.75	57.49	57.49	114.98
Tetrachloroethylene	3.63	3.63	2.54	3.63	2.54	3.63	3.63	1.81
**Chronic daily intake (μg kg^−1^ day^−1^)**								
Benzene	0.001	0.010	0.010	0.027	0.001	0.001	0.001	0.012
Toluene	0.045	0.301	0.988	0.892	0.011	0.001	0.010	0.412
Ethylbenzene	0.654	0.476	0.179	2.112	0.046	0.001	0.128	0.087
Xylene	0.803	1.982	0.771	2.112	0.189	0.001	0.122	0.395
Styrene	0.001	0.001	0.001	0.002	0.001	0.001	0.001	0.002
Dichlorofluoromethane	0.002	0.002	0.002	0.003	0.001	0.001	0.098	0.003
Acetone	0.001	0.090	0.005	0.122	0.014	0.001	0.001	0.002
n-Hexane	0.001	0.001	0.002	0.002	0.001	0.001	0.001	0.002
n-Heptane	0.001	0.001	0.001	0.002	0.001	0.001	0.001	0.002
n-Octane	0.001	0.001	0.001	0.002	0.006	0.001	0.001	0.002
n-Nonane	0.001	0.001	0.001	0.002	0.007	0.001	0.001	0.002
n-Decane	0.001	0.001	0.001	0.002	0.001	0.001	0.001	0.002
n-Butylacetate	0.001	0.001	0.001	0.001	0.001	0.001	0.001	0.001
Trichloroethylene	0.001	0.001	0.001	0.002	0.001	0.001	0.001	0.002
Tetrachloroethylene	0.001	0.001	0.001	0.001	0.001	0.001	0.001	0.001

**Table 5:** Lifetime risk of cancer (LTCR) of the characterized VOCs.

ELCR	OEM Salon	Production Salon-PC	CED Production	Washing Salon-PC	OEM Lab	PC Lab	Dispatch	CED Topcoat
Benzene	3.97 × 10^−5^	2.78 × 10^−4^	2.98 × 10^−4^	7.94 × 10^−4^	1.99 × 10^−5^	3.97 × 10^−5^	3.97 × 10^−5^	3.37 × 10^−4^
Ethylbenzene	1.64 × 10^−3^	1.19 × 10^−3^	4.47 × 10^−3^	5.28 × 10^−2^	1.14 × 10^−4^	2.33 × 10^−6^	3.21 × 10^−3^	2.19 × 10^−4^
Trichloroethylene	1.27 × 10^−5^	1.27 × 10^−5^	1.27 × 10^−5^	2.53 × 10^−5^	6.33 × 10^−6^	1.27 × 10^−5^	1.27 × 10^−5^	2.53 × 10^−5^
Tetrachloroethylene	2.91 × 10^−5^	2.91 × 10^−5^	2.03 × 10^−5^	2.91 × 10^−5^	2.03 × 10^−5^	2.91 × 10^−5^	2.91 × 10^−5^	1.45 × 10^−5^
Styrene	5.2 × 10^−7^	5.2 × 10^−7^	5.2 × 10^−7^	1.04 × 10^−6^	4.68 × 10^−7^	5.2 × 10^−7^	4.68 × 10^−7^	1.04 × 10^−6^
Total LTCR	1.15 × 10^−3^	8.93 × 10^−4^	3.77 × 10^−4^	3.85 × 10^−3^	8.87 × 10^−5^	1.8 × 10^−5^	2.4 × 10^−4^	2.28 × 10^−4^

## Data Availability

Not applicable.
